# Mental distress in a clinical nurse due to a false‐positive COVID‐19 antibody test result during the COVID‐19 epidemic in Japan: A case report

**DOI:** 10.1002/ccr3.4122

**Published:** 2021-05-15

**Authors:** Yuzo Shimazu, Yurie Kobashi, Tianchen Zhao, Yositaka Nishikawa, Toyoaki Sawano, Akihiko Ozaki, Daiji Obara, Masaharu Tsubokura

**Affiliations:** ^1^ Department of Radiation Health Management Fukushima Medical University School of Medicine Fukushima City Japan; ^2^ Department of General Internal Medicine Hirata Central hospital Hirata, Ishikawa district, Fukushima Japan; ^3^ Department of Surgery Jyoban Hospital of Tokiwa Foundation Iwaki, Fukushima Japan; ^4^ Department of Breast Surgery Jyoban Hospital of Tokiwa Foundation Iwaki, Fukushima Japan

**Keywords:** COVID‐19 antibody test (qualitative antibody kit), COVID‐19 epidemic, false positive, mental distress

## Abstract

This study suggests the importance of instituting accompanying measures to prevent potential negative mental and social impacts on people receiving false‐positive results.

## Graphical abstract

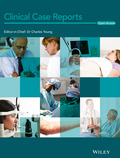

## 1 INTRODUCTION

As coronavirus antibody testing is simpler than performing polymerase chain reaction, it is being actively developed and sold. However, few mental problems associated with false‐positive testing results have been reported. We describe the case of a nurse who suffered from mental distress due to a false‐positive antibody test result.

The coronavirus disease 2019 (COVID‐19) pandemic has been ongoing since the first case occurred in Wuhan, China, in November 2019. Since then, the number of COVID‐19 infections and deaths has been increasing worldwide. Although various infection control measures have been implemented, new infections remain difficult to prevent. By the end of September 2020, the number of people infected with COVID‐19 worldwide reached 36 million, while the number of deaths exceeded 1 050 000.^1^ Hence, further precautions are necessary to control the pandemic.

Antibody testing is useful in evaluating and controlling infections, assessing the effects of new vaccines, and as a marker of severity of SARS‐CoV‐2.^2^, ^3^, ^4^, ^5^ As rapid immunochromatographic (ICG) testing kits are easy to use and require no apparatus, they are used widely. Hence, increasing the usage and improving the accuracy of these tests have recently become vital public health issues.^6^ Antibody testing is categorized as qualitative and quantitative; the former tests have been widely developed and sold globally. Each product has suboptimal sensitivity and specificity in detecting COVID‐19 infection, and a definitive diagnosis cannot be made based solely on these tests; furthermore, the accuracy of antibody testing has been frequently questioned. Currently, little information is available on the mental health impact of these tests, including the psychological effects of false‐positive testing results.

We herein report the case of a Japanese healthcare worker, who suffered from severe mental distress due to a false‐positive COVID‐19 antibody test result. Discussing the effects of false‐positive antibody testing results may provide crucial information to clinicians, who are considering to expand the application of COVID‐19 antibody testing.

### 1.1 History of the new coronavirus antibody testing in our hospital

In mid‐April 2020, the Seireikai Health Care Group introduced a new COVID‐19 antibody qualitative testing kit manufactured by Vazyme Co., Ltd. The sensitivity and specificity of this product were 91.54% (95% CI: 86.78%‐94.65%) and 97.02% (95% CI: 94.74%‐98.33%), respectively.^7^ Our group measured the IgM and IgG antibodies of medical staff (excluding clerical staff) working in the office. A polymerase chain reaction (PCR) assay was performed in 51 participants, whose results indicated IgM positivity on rapid ICG testing; all PCR test results were negative. None of the staff had any subjective symptoms related to COVID‐19 during the survey period. The doctor in charge of the testing explained the process and results of the testing to the staff who tested positive on qualitative antibody testing. Two months after the survey, none of the participants were diagnosed with COVID‐19. Thus, the positive IgM results were determined to be false positives.

The clinic where the reported member of staff worked was located in a mountainous area, which is approximately 40 minutes away from the major cities of the Naka‐dori region, Fukushima Prefecture, by car. As of 1 July 2020, the prevalence of the aging population in this region was 39.7%, which is much higher than that of Japan (28%).^8^, ^9^ As of 30 September 2020, the number of new coronavirus infections in Fukushima Prefecture was 279, and transmission had not been confirmed. A total of 40 000 people live in this area, which is served by a 200‐bed hospital and clinic as well as several nursing care and welfare facilities (including day care rehabilitation facilities).

## 2 CASE

This patient was a member of the medical staff, who tested positive on IgM qualitative antibody testing. The patient was a woman in her 40s, who worked as a nurse in charge of the outpatient department; her family lived in a town near the hospital. Before taking the antibody test, she had no history of close contact with infected patients and had no obvious cold‐like symptoms (fever, cough, and sore throat, among others). She also had no medical history of note. She only presented with irritation, which could be a possible presenting feature of COVID‐19 infection. However, she did not have any atypical symptoms.^10^, ^11^

As COVID‐19 infection was not ruled out, additional PCR testing was performed. After being notified of the positive qualitative antibody testing results, she became depressed and experienced trouble with sleeping. The husband also stayed at home until he was allowed to return to work. She and her husband were concerned about the negative effects of becoming the source of an outbreak; these included harassment or slander, rumors, and identification of her personal information by the press. Moreover, she suspected that she would be denied to work in her workplace and feared that her children would be bullied. After careful reflection, she recollected that she had no fever since approximately 2 weeks prior, but experienced very mild symptoms of irritation in her throat. As she thought it was a possibly COVID‐19 symptom, she experienced feelings of guilt; she felt that medical professionals should not become the source of infection. She also feared that the clinic she worked at could be shut down due to transmission of the virus. Once her negative PCR testing results were revealed, the doctor explained the testing results, as well as the follow‐up system in detail. Her senior and colleagues were involved in preparing online medical treatment approaches to support her mentally. In addition, she had informed her husband that he had no positive test results or the possibility of exposure at home. He therefore accepted the situation and supported her mentally. The patient was able to regain her sense of security and confidence in her subsequent work, and her subjective psychological symptoms disappeared. After 3 days, she was allowed to return to work.

## 3 DISCUSSION

In this report, we describe the case of a nurse who suffered from mental distress following a positive COVID‐19 antibody testing result. Despite significant efforts to prevent infection, the COVID‐19 antibody testing result was positive; this resulted in feelings of helplessness and loss of self‐confidence.

This report suggests the importance of instituting accompanying measures to prevent potential negative mental impacts on people receiving false‐positive antibody testing results. The present patient was a nurse, who comes into contact with a large number of patients at the clinic daily. In some countries where COVID‐19 outbreaks have already occurred,^12^ healthcare systems have collapsed, endangering the mental health of medical staff. In particular, a survey conducted in Wuhan, China, with approximately 800 medical staff as participants (approximately 60% of whom worked in hospitals) showed that 50% experienced depressive symptoms, followed by anxiety and insomnia.^13^ Previous studies have also reported on several psychological effects that the coronavirus pandemic has had on medical staff.^14^, ^15^, ^16^, ^17^ While these reports have focused on medical professionals involved in the treatment of actually infected patients, this case is significant as it demonstrates the psychological effects caused by false‐positive results. Generally, false‐positive results have been shown to have strong adverse psychological effects, as with HIV screening.^18^, ^19^ Currently, PCR testing is recognized as the golden standard for diagnosing COVID‐19; antibody testing has not advanced adequately to reliably provide a diagnosis on its own. In the present case, the patient suffered from the mental burden of an actual diagnosis of COVID‐19. While systemic antibody tests may be used to determine the effectiveness of vaccines, and as markers for aggravation, cumulative knowledge regarding false positives needs to be acquired in future. In this case, we diagnosed the patient as having depression based on clinical symptoms.

The present case highlights mental distress associated with the possible social impact of COVID‐19 infection. First, the present patient was concerned about becoming the source case of the outbreak in an area where COVID‐19 has not been introduced. In regions where the population has declined in recent years, anxiety related to the spread of gossip among neighboring residents could be an issue. In a rural area of Japan, children whose parents work in healthcare facilities were reportedly bullied or discriminated against at school.^20^ This prejudice and discrimination, caused by stigma, further exacerbates the mental health burden and increases the fear of COVID‐19 infections among medical staff. Consequently, there have been reports of suicide. Second, the present patient felt guilty about bringing the infection to a hospital where it should be prevented and about the increasing burden on other medical staff due to her infection. Although this feeling of guilt may result from the fact that it is the responsibility of the medical staff to treat the patient, it could also be related to the feeling that medical staff will be accused of becoming infected. Third, the present patient was worried that her infection could disrupt her home environment.^21^, ^22^, ^23^ In some European countries, including Spain and Portugal, the percentage of male nurses exceeds 20%.^24^ In contrast, female nurses account for 92% of all nurses in Japan; however, the proportion of male nurses is gradually increasing, and their overall number has doubled in the past 10 years.^25^ Thus, female nurses play important roles in performing household chores in Japan, where the social environment places a considerable burden of the housework on women; this may have caused such feelings. Concerns regarding this social impact may have contributed to the further exacerbation of mental distress. It is essential to prepare in advance for such possible social impacts and to have a social system where the impact caused by infection is not considered to be the responsibility of the individual.

In conclusion, this study suggests the importance of instituting accompanying measures to prevent potential negative mental and social impacts on people receiving false‐positive antibody testing results. As COVID‐19 may threaten humanity repeatedly, establishing physical, mental, and social support systems for medical personnel involved in treating and caring for these patients will be necessary in the future.

## CONFLICT OF INTEREST

The authors declare no competing interests.

## AUTHOR CONTRIBUTIONS

YS and MM: drafted the manuscript. YK, TZ, YN, TS, AO, and DO: helped to draft the manuscript. DO, YK, and MT: played a central role in providing medical care at this clinic. All authors read and approved the final manuscript.

## INFORMED CONSENT

Written informed consent for the publication of patient data was obtained from the patient.

## Data Availability

The authors confirm that the data supporting the findings of this study are available within the article.
